# Atlas and Essentials of Bacteriology

**Published:** 1898-01

**Authors:** 


ATLAS AND ESSENTIALS OF BACTERIOLOGY. BY PROF. K. B. LEHMANN, Würzburg and Dr. Rudolf Neumann, Würzburg. Sixty-three chromo-lithographic plates, comprising 558 separate figures, with explanatory text facing plates and 128-page treatise. Illustrated. New York: William Wood & Co. 1897.

This volume is similar in size, appearance and general arrangement to those issued during the past year—namely, on ophthalmology, nervous diseases, fractures and dislocations, obstetrics and gynecology. The full set, making a handsome addition to a physician's library, may be obtained for $15.

The volume in question is edited by two of the leading bacteriologists of Germany and gives, as no other work can for the money, a complete and accurate pictorial portrayal of the various known microörganisms, both the culture and the microscopical appearance. The volume contains sixty-three chromo-lithographic plates, comprising 558 figures, and numerous engravings. The text, 135 pages, is devoted to the methods employed in the bacteriological laboratory, the description of the various bacteria and a carefully-prepared index.

The volume is a handsome illustration of the bookmaking and chromo-lithographic art. W. C. K.

